# Dual similarity solutions of MHD stagnation point flow of Casson fluid with effect of thermal radiation and viscous dissipation: stability analysis

**DOI:** 10.1038/s41598-020-72266-2

**Published:** 2020-09-21

**Authors:** Liaquat Ali Lund, Zurni Omar, Ilyas Khan, Dumitru Baleanu, Kottakkaran Sooppy Nisar

**Affiliations:** 1 School of Quantitative Sciences, Universiti Utara Malaysia, 06010 Sintok, Kedah Malaysia; 2 KCAET Khairpur Mirs, Sindh Agriculture University, Tandojam Sindh, 70060 Pakistan; 3 Faculty of Mathematics and Statistics, Ton Duc Thang University, Ho Chi Minh City, Vietnam; 4 Department of Mathematics, Cankaya University, Ankara, Turkey; 5 Institute of Space Sciences, 077125 Magurele, Romania; 6 Department of Medical Research, China Medical University Hospital, China Medical University, Taichung, Taiwan; 7 Department of Mathematics, College of Arts and Sciences, Prince Sattam Bin Abdulaziz University, Wadi Al Dawasir, 11991 Saudi Arabia

**Keywords:** Mechanical engineering, Mathematics and computing, Nanoscience and technology

## Abstract

In this paper, the rate of heat transfer of the steady MHD stagnation point flow of Casson fluid on the shrinking/stretching surface has been investigated with the effect of thermal radiation and viscous dissipation. The governing partial differential equations are first transformed into the ordinary (similarity) differential equations. The obtained system of equations is converted from boundary value problems (BVPs) to initial value problems (IVPs) with the help of the shooting method which then solved by the RK method with help of maple software. Furthermore, the three-stage Labatto III-A method is applied to perform stability analysis with the help of a bvp4c solver in MATLAB. Current outcomes contradict numerically with published results and found inastounding agreements. The results reveal that there exist dual solutions in both shrinking and stretching surfaces. Furthermore, the temperature increases when thermal radiation, Eckert number, and magnetic number are increased. Signs of the smallest eigenvalue reveal that only the first solution is stable and can be realizable physically.

## Introduction

Two significant classes of fluids have gotten a lot of consideration from researchers and mathematicians in the previous few years, specifically Newtonian and non-Newtonian fluids. A fluid in which the rate of change of deformation is directly proportionate to viscous stresses is known as Newtonian fluid. On contrary, a fluid wherein properties of the fluid are not the same as Newtonian fluid is known as non-Newtonian fluid. The important property of non-Newtonian fluid is viscosity. In many industrial problems, the comportment of non-Newtonian fluids is more important as compared to Newtonian fluids^1,2^. Some applications of non-Newtonian fluids can be seen in polymer engineering, manufacturing of foods, petroleum drilling, certain separation processes, and papers^3,4^. It is very hard to convey all properties of numerous non-Newtonian fluids in a single momentum equation due to non-linearity among the rate of strain and stress of the fluids. In this regard, many non-Newtonian models have been proposed based on variation of physical characteristics^5–9^. Among these models, Casson fluid model which can be defined as “a shear-thinning fluid in which zero viscosity at an infinite rate of shear and an infinite viscosity at zero rates of shear”^10^ is the most popular one. Ullah et al.^11^ inspected Casson fluid over a non-linear stretching cylinder and found that the shear stress is directly proportional to suction/blowing and porosity parameter. Dual solutions have been obtained by Yahaya et al.^12^ in Casson fluid with the effect of homogeneous-heterogeneous reactions. Moreover, the analysis of the stability was carried out to determine which solution is stable. They found that only the first solution is stable and can be easily utilized in different applications. Khan and Husain^13^ explored Casson liquid on the circle disk where the system of governing ordinary differential equations was solved by utilizing homotopy method. Meanwhile, Hamid et al.^14^ considered Casson liquid encased in the cavity. Further, double solutions were found by Hamid et al.^15^ within the sight of thermal radiation. Stability analysis was additionally performed by utilizing of BVP4C in the MATLAB programming. Some ongoing improvements on Casson fluid can be found in these articles^16–20^.

The study of boundary layer Casson fluid flow on stretching and shrinking surfaces was widely investigated for single solution cases. Stretching surface has many applications in many manufacturing such as the extrusion of the molten polymers due to the slit die in productions of plastic sheets, paper productions, wires as well as in the fibers coating process of the food stuff. The qualities of final products in such processes depend heavily upon the cooling rate in the process of the heat exchange. Therefore, a MHD parameter is an important element to be considered so that the rate of the cooling can be controlled in order to obtain the desired quality products. Crane^21^ proposed a method for solving incompressible steady-state 2-D viscous fluid on which later extended to many diverse aspects. Some of its recent important directions over-stretching flows can be seen in these articles^22–26^. Vajravelu^27^ and Cortell^28^ considered viscous fluid on a non-linear stretching sheet. In this paper, we also considered a stretching surface with the shrinking surface due to its extensive utilizations in different fields.

From the previous couple of years, multiple similarity solutions of fluid flow problems have been considered on the stretching and shrinking sheets in the presence and absence of bouncy effect by numerous scholars^29–32^. These multiple solutions exist due to several physical parameters impacts such as suction parameter, mixed convection parameter and so forth. Furthermore, the past researches demonstrated the probabilities of the occurrence of multiple similarity solutions of fluid flow over a shrinking sheet are more than over a stretching surface^31^. The possibilities of non-uniqueness solution of fluid flow on a stretching surface are probable when the flow is stagnation point flow or opposing flow. Similarly, multiple solutions for Newtonian fluids can be gotten easily as compared to non-Newtonian fluids. It is stated in the previously published literature that non-uniqueness of the solutions occurs because of the existence of non-linearity in the fluid equations^29,30^. However, the models of non-Newtonian fluids contain many non-linear terms and thus leads to non-existence of multiple solutions.

The boundary layer flows of fluid and their similarity solutions are gotten much attention due to their vast applications in many industrial fields^32^. In real situations, multiple solutions cannot be visualized in the boundary layer problems and difficult to be detected. Hence, many researchers fail to notice multiple solutions^33,34^. Multiple solutions of MHD fluid flow problems have been examined theoretically as well as numerically by numerous researchers. Dero et al.^35^ obtained triple solutions during the investigation of micropolar fluid with thermal radiation effect. Further, Dero et al.^36^ inspected the unsteady flow of nanofluid on the shrinking sheet and found double solutions in deaccelerated case. It seems that Ridha and Curie^37^ are the pioneers who found dual solutions in the opposing flow. The motivation behind this study is to consider multiple similarity solutions of the MHD stagnation point flow of Casson fluid on a permeable exponentially stretching and shrinking surfaces unanimously with viscous dissipation and thermal radiation effect.

## Mathematical formulation

**Figure 1:**
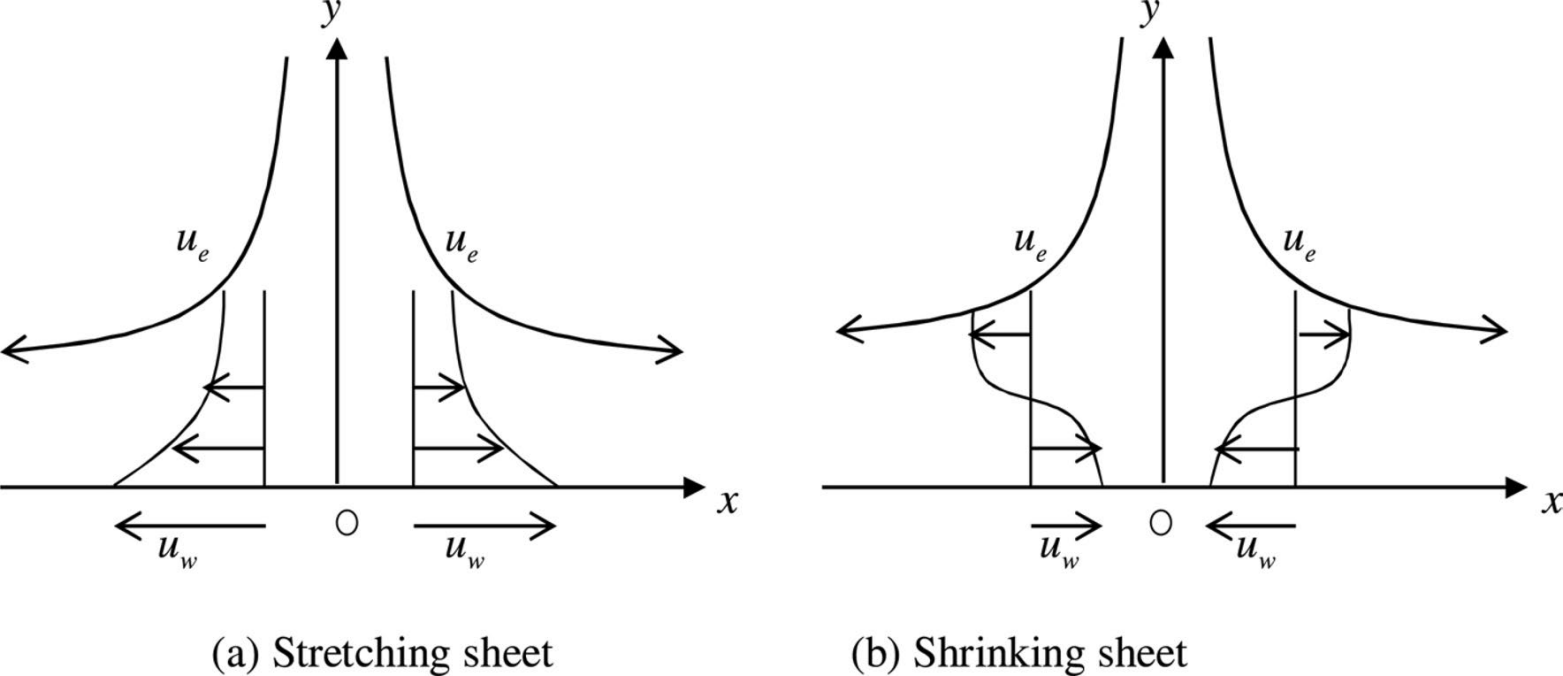
Physical model and coordinate system.

There has been studied as steady incompressible 2-D stagnation point flow of Casson electrically leading fluid on an exponentially shrinking and stretching surfaces with the impact of thick viscous dissipations and thermal radiation. There has additionally been supposed that rheological equation of the state for the isotropic and the incompressible progression of the Casson fluid which are reported as (allude^10^):

1 $${\tau }_{ij}=\left\{\begin{array}{l}\left({\mu }_{B}+\left(\frac{{P}_{y}}{\sqrt{2\pi }}\right)\right)2{e}_{ij}, \pi >{\pi }_{c} \\ \left({\mu }_{B}+\left(\frac{{P}_{y}}{\sqrt{2{\pi }_{c}}}\right)\right)2{e}_{ij}, \pi <{\pi }_{c} \end{array}\right. $$

where the plastic dynamic viscosity of the non-Newtonian fluid is meant by ${\mu }_{B}$, $\pi$ signifies the result of deformation rate segment, that is, , π = ${e}_{ij}{e}_{ij}$ is (i, j)th deformation rate part and ${\pi }_{c}$ is critical value of the $\pi$ which depends on the non-Newtonian model, and ${P}_{y}$ indicates the yield stress of the fluid. Moreover, a system of cartesian coordinate is taken into account, where the *x*-axis is supposed alongside the shrinking/Stretching surface and the *y*-axis is normal to it. Further, ${u}_{w}=a{ e}^{x/l}$ is the shrinking and stretching velocity of surface. The uniform magnetic field is applied to the normal of the fluid flow *B*=${B}_{0}{e}^{x/2l}$ where ${B}_{0}$ is the constant magnetic strength (Fig. 1). The field of induced magnetic is disregarded as a result of the small estimation of the magnetic Reynolds number. With the above assumptions, we get following equations

2 $$\frac{\partial u}{\partial x}+\frac{\partial v}{\partial y}=0$$

3 $$u\frac{\partial u}{\partial x}+v\frac{\partial u}{\partial y}={u}_{e}\frac{\partial {u}_{e}}{\partial x}+\vartheta \left(1+\frac{1}{\beta }\right)\frac{{\partial }^{2}u}{\partial {y}^{2}}+\frac{\sigma {B}^{2}}{\rho }\left({u}_{e}-u\right)$$

4 $$u\frac{\partial T}{\partial x}+v\frac{\partial T}{\partial y}=\left( \alpha +\frac{16{\sigma }_{1}{T}_{\infty }^{3}}{3{K}^{*}\rho {c}_{p}}\right)\frac{{\partial }^{2}T}{\partial {y}^{2}}+\frac{\mu }{\rho {c}_{p}}\left(1+\frac{1}{\beta }\right){\left(\frac{\partial u}{\partial y}\right)}^{2} $$

along the following boundary conditions

5 $$\begin{array}{c}v={v}_{w} , u=\lambda {u}_{w}, T={ T}_{w} \,\, \mathrm{at} \,\, y=0\\ u \to {u}_{e}= b{e}^{x/l}, T \to {T}_{\infty } \,\, \mathrm{as } \,\, y \to \infty \end{array}$$

where ${T}_{w}={T}_{\infty }+{T}_{0}{e}^{\frac{x}{2\mathcal{l}}}$ is the temperature of wall, ${v}_{w}=- \sqrt{\frac{{\vartheta U}_{w}}{2l}}{e}^{x/2l} S$ where *S* is the suction/injunction parameter, and ${u}_{e}=b{ e}^{x/l}$ is the stagnation point.

Following similarity variables are used to get the similarity solutions

6 $$\psi =\sqrt{2\vartheta la}{e}^{x/2l}f\left(\eta \right) , \theta (\eta )=\frac{\left({T-T}_{\infty }\right)}{\left({T}_{w}{- T}_{\infty }\right)}, \eta =y\sqrt{\frac{a}{2\vartheta l}} {e}^{x/2l}$$

and $\psi$ is the stream function and can be written in velocity component as follows,

7 $$u = \frac{\partial \psi }{\partial y} , v = - \frac{\partial \psi }{\partial x}$$

By employing Eqs. (6) and (7) in Eqs. (2)–(5), we obtained

8 $$\left(1+\frac{1}{\beta }\right){f}^{\prime\prime\prime}+f{f}^{\prime\prime}-2{f}^{\prime 2}+2{A}^{2}-M\left({f}^{{\prime}}-A\right)=0$$

9 $$\frac{1}{Pr}\left(1+\frac{4}{3}{R}_{d}\right){\theta }^{\prime\prime}+f{\theta }^{{\prime}}-{f}^{{\prime}}\theta +Ec.\left(1+\frac{1}{\beta }\right){\left({f}^{\prime\prime}\right)}^{2} =0$$

along boundary conditions

10 $$\left\{\begin{array}{c}f\left(0\right)=S , {f}^{{\prime}}\left(0\right)=\lambda , \theta \left(0\right)=1\\ {f}^{{{\prime}}}\left(\eta \right) \to A, \theta \left(\eta \right)\to 0 as \eta \to \infty \end{array}\right.$$

where $M=\frac{2l\sigma {\left({B}_{0}\right)}^{2}}{\rho a}$ is the Hartmann number, $Pr=\frac{\vartheta }{\alpha }$ denotes the Prandtl number, ${R}_{d}=\frac{4{\sigma }_{1}{T}_{\infty }^{3}}{k{K}^{*}}$ is the thermal radiation, $Ec=\frac{{u}_{w}^{2}}{\left({T}_{w}-{T}_{\infty }\right){c}_{p}}$ is Eckert number, $\lambda$ is the stretching and shrinking parameter, and $A=\frac{b}{a}$ is the velocity ratio parameter of the stagnation point.

Physical quantities of interests are coefficient of skin friction, and the local Nusselt number, which are reported as:

11 $${C}_{f}=\frac{{\left[\left(1+\frac{1}{\beta }\right)\frac{\partial u}{\partial y}\right]}_{y=0}}{\rho {U}_{w}^{2}}; {N}_{u}=\frac{-x{\left(\frac{\partial T}{\partial y}\right)}_{y=0}}{\left({T}_{w}{- T}_{\infty }\right)}$$

12 $${C}_{f}{\left({Re}_{x}\right)}^\frac{1}{2}\sqrt{\frac{2l}{x}}=\left(1+\frac{1}{\beta }\right){f}^{\prime\prime}\left(0\right);{N}_{u}{\left({Re}_{x}\right)}^{-\frac{1}{2}}=-\left(1+\frac{4}{3}{R}_{d}\right){\theta }^{{\prime}}\left(0\right)$$

## Stability analysis

In this study, we found dual solutions of Eqs. (8)–(9) along with the boundary conditions (10) for both surfaces. Thus, it is needed to do the stability analysis to detect a stable solution which can be physically reliable after the time passes. According to Hamid et al.^15^, the upper branch solutions always show stability and physically reliability. On the other hand, the second solutions are unstable and consequently physically unreliable. The same remarks were reported by many researchers [refer to^38–42^].

In order to accomplish the solutions’ temporal stability, the unsteady form of Eqs. (3)–(4) must be considered by proposing the new dimensionless time variable $\tau$ where $\tau$ is related to solutions of Eqs. (8)–(9). Equations (3)–(4) can be expressed for the unsteady state flow as follows

13 $$\frac{\partial u}{\partial t}+u\frac{\partial u}{\partial x}+v\frac{\partial u}{\partial y}={u}_{e}\frac{\partial {u}_{e}}{\partial x}+\vartheta \left(1+\frac{1}{\beta }\right)\frac{{\partial }^{2}u}{\partial {y}^{2}}+\frac{\sigma {B}^{2}}{\rho }\left({u}_{e}-u\right)$$

14 $$\frac{\partial T}{\partial t}+u\frac{\partial T}{\partial x}+v\frac{\partial T}{\partial y}=\left( \alpha +\frac{16{\sigma }_{1}{T}_{\infty }^{3}}{3{K}^{*}\rho {c}_{p}}\right)\frac{{\partial }^{2}T}{\partial {y}^{2}}+\frac{\mu }{\rho {c}_{p}}\left(1+\frac{1}{\beta }\right){\left(\frac{\partial u}{\partial y}\right)}^{2}$$

The new dimensionless time-dependent variable $\tau =\frac{a}{2l}{e}^{x/l}.t$ is presented. Therefore, Eq. (6) can be written as follows:

15 $$\psi =\sqrt{2\vartheta la}{e}^{x/2l}f\left(\eta ,\tau \right) , \theta (\eta ,\tau )=\frac{\left({T-T}_{\infty }\right)}{\left({T}_{w}{- T}_{\infty }\right)}, \eta =y\sqrt{\frac{a}{2\vartheta l}} {e}^{x/2l},\tau =\frac{a}{2l}{e}^{x/l}.t$$

By substituting Eq. (15) in Eqs. (13)–(14), we get

16 $$\left(1+\frac{1}{\beta }\right)\frac{{\partial }^{3}f}{\partial {\eta }^{3}} + f \frac{{\partial }^{2}f}{\partial {\eta }^{2}}-2{\left(\frac{\partial f}{\partial \eta }\right)}^{2}+2{A}^{2}-M\left(\frac{\partial f}{\partial \eta }-A\right)-\frac{{\partial }^{2}f\left(\eta , \tau \right)}{\partial \tau \partial \eta }=0$$

17 $$\frac{1}{Pr}\left(1+\frac{4}{3}Rd\right)\frac{{\partial }^{2}\theta }{\partial {\eta }^{2}} + f\frac{\partial \theta }{\partial \eta }-\frac{\partial f}{\partial \eta }\theta +\mathrm{Ec}\left(1+\frac{1}{\beta }\right){\left(\frac{{\partial }^{2}f}{\partial {\eta }^{2}}\right)}^{2}-\frac{\partial \theta }{\partial \tau }=0$$

With corresponding boundary conditions

18 $$\left\{\begin{array}{c}f\left(0,\tau \right)=S; \frac{\partial f\left(0, \tau \right)}{\partial \eta }=\lambda ; \theta \left(0,\tau \right)=1\\ {f}^{{{\prime}}}\left(\eta ,\tau \right) \to A, \theta \left(\eta ,\tau \right)\to 0 as \eta \to \infty \end{array}\right.$$

According to Lund et al.^43^, Ismail et al.^44^, and Naganthran et al.^45^, the stability of the dual solutions is tested by perturbing the steady solution by using following functions

19 $$\left\{\begin{array}{c}f\left(\eta ,\tau \right) ={f}_{0}\left(\eta \right) + {e}^{-\tau } {F}_{0}\left(\eta \right)\\ \theta \left(\eta ,\tau \right) = {\theta }_{0}\left(\eta \right) + {e}^{-\tau } {G}_{0}\left(\eta \right)\end{array}\right.$$

where corresponding small relatives of ${f}_{0}\left(\eta \right)$ and ${\theta }_{0}\left(\eta \right)$ are ${F}_{0}\left(\eta \right)$ and ${G}_{0}\left(\eta \right)$ and unknow eigenvalue is γ. It is worth to mention that perturbated function has been considered in the form of exponential as compared to power function as these functions increase and decrease more rapidly as compared to the power functions. By putting Eq. (19) into Eqs. (16–17), we get linearized eigenvalue problem as follows:

20 $$\left(1+\frac{1}{\beta }\right){F}_{0}^{\prime\prime\prime}+{f}_{0}\left(\eta \right){F}_{0}^{\prime\prime}+{F}_{0}{f}_{0}^{\prime\prime}-4{f}_{0}^{{\prime}}{F}_{0}^{{\prime}}-M{F}_{0}^{{\prime}}+\gamma{F}_{0}^{{\prime}}=0$$

21 $$\frac{1}{Pr}\left(1+\frac{4}{3}Rd\right){G}_{0}^{\prime\prime}+{f}_{0}{G}_{0}^{{\prime}}+{F}_{0}{\theta }_{0}^{{\prime}}-{f}_{0}^{{\prime}}{G}_{0}-{F}_{0}^{{\prime}}{\theta }_{0}+2Ec\left(1+\frac{1}{\beta }\right){f}_{0}^{\prime\prime}{F}_{0}^{\prime\prime}+\gamma{G}_{0}=0$$

Subject to boundary conditions

22 $$\left\{\begin{array}{c}{F}_{0}\left(0\right) = 0, {F}_{0}^{{\prime}}\left(0\right) =0, {G}_{0}(0) =0\\ {F}_{0}^{{\prime}}(\eta ) \to 0, {G}_{0}(\eta ) \to 0 as \eta \to \infty \end{array}\right.$$

System of linearized eigenvalue problem Eqs. (20)–(21) along boundary conditions (22) is solved and obtained the infinite set of eigenvalues $\left({\gamma }_{1}<{\gamma }_{2}<{\gamma }_{3}<\cdots\right)$.

The solution is said to be stable flow if and only if the sign of the ${\gamma }_{1}$ is positive which shows the initial decay, as time passes. On the other hand, if the sign of the ${\gamma }_{1}$ is negative, at that point the flow solution shows the initial growth of development and the solution is said to be an unstable solution, as time passes.

## Result and discussion

In this segment of the article, we discuss about the effect of numerous physical rising parameters on temperature, velocity, rate of heat transfer, and coefficient of skin friction profiles. In order to validate the results of our numerical technique, the numerical results of $-\left(1+\frac{1}{\beta }\right){f}^{\prime\prime}\left(0\right)$ were compared with results obtained by Hussain et al.^46^ for different values of the Casson parameter $\beta$ and magnetic parameter $M$ as given in Table 1. The results are in the excellent agreement which indicate that our method is reliable. Table 2 was constructed for the values of smallest eigenvalue ${\gamma }_{1}$ for various values of the velocity ratio parameter.

**Table 1:** Values of $-\left(1+\frac{1}{\beta }\right){f}^{\prime\prime}\left(0\right)$ for different values of $\beta$ and $M$ where $A=0,M={M}^{2}, \lambda =1,$ and $S=0$.

$\beta$	$M$	Hussain et al.^46^	Present results
		$$-\left(1+\frac{1}{\beta }\right){f}^{\prime\prime}\left(0\right)$$	
0.7	0.5	2.146677	2.146676800
1.2	0.5	1.865142	1.865142292
1.2	0.0	1.735577	1.735580976
1.2	0.4	1.819679	1.819679224
1.2	0.7	1.980908	1.980908405

**Table 2:** The values of the smallest eigenvalue $${\gamma }_{1}$$ for different values of S and $$\lambda $$ where $$\beta =1.5,$$$$M=0.25, A=0.1, Pr=1, Ec=0.1$$.

*S*	$\lambda$	${\gamma }_{1}$	
		1st solution	2nd solution
3	0.5	1.27921	− 1.5514
3	− 0.5	0.85147	− 0. 9,058
2.5	0.5	1.0572	− 1.2417
2.5	− 0.5	0.80362	− 0.8725
2.25	0.5	0.92832	− 1.0562
2.25	− 0.5	0.6825	-0.8386

**Figure 2:**
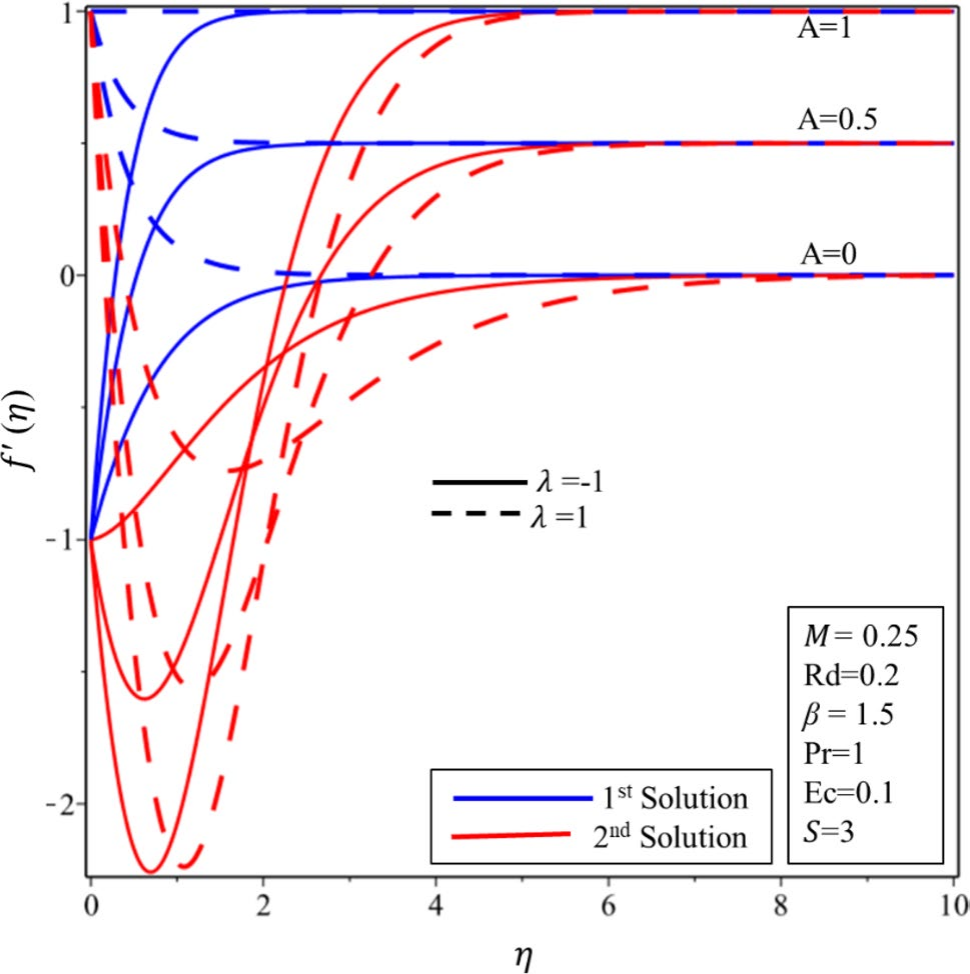
Velocity profile for different values of *A.*

**Figure 3:**
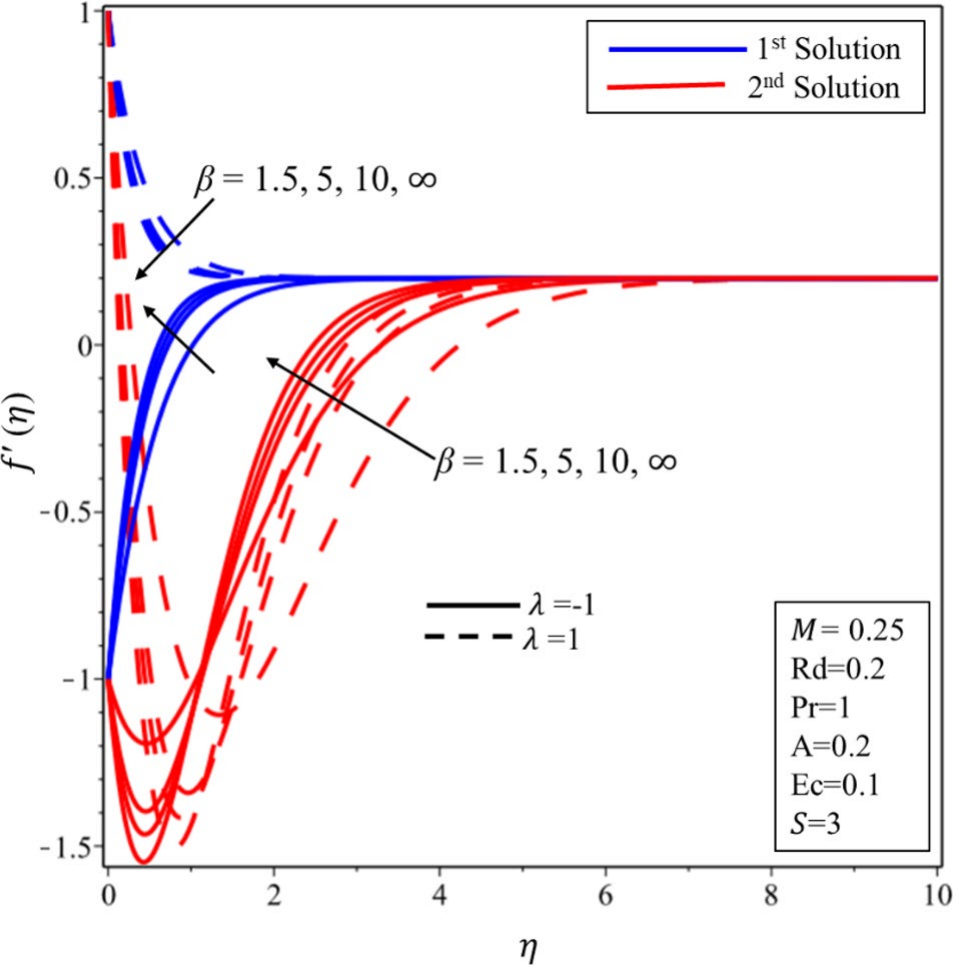
Velocity profile for different values of $\beta$*.*

**Figure 4:**
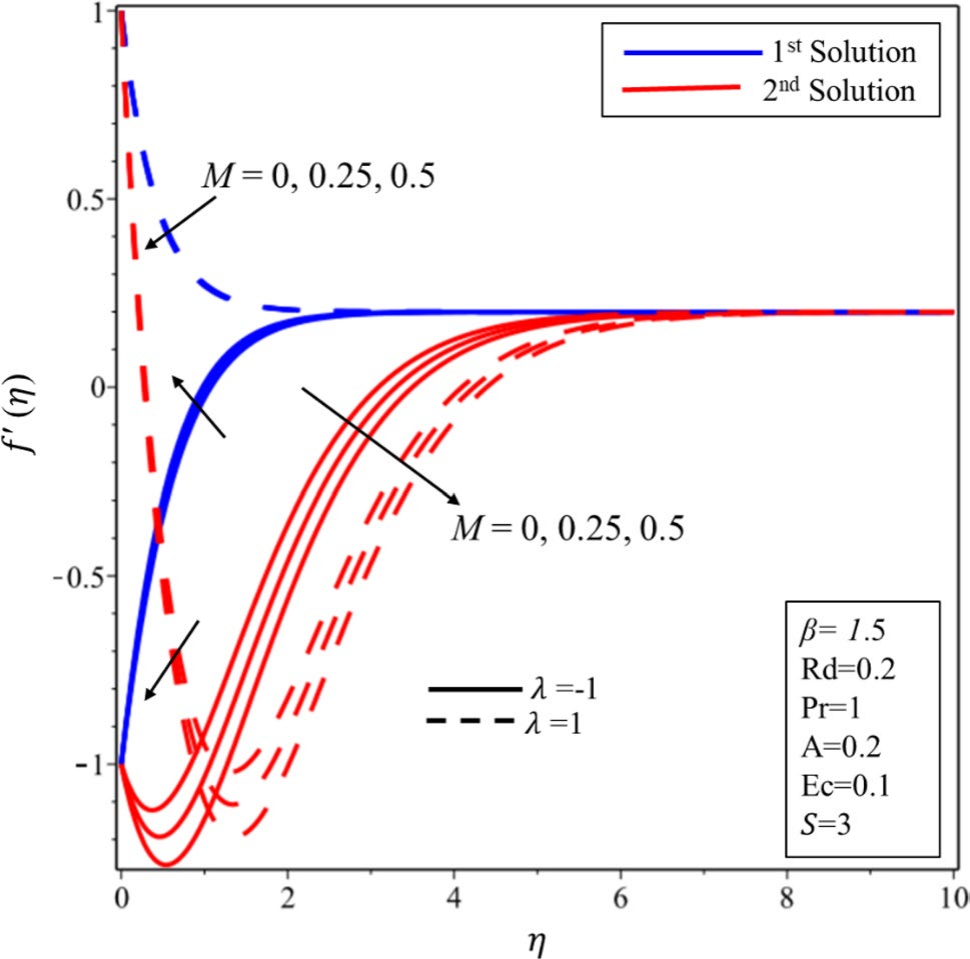
Velocity profile for different values of $M$*.*

Figure 2 exhibits the profile of velocity for numerous estimations of the stagnation point $A$ in both shrinking and stretching surfaces. It is worth to note that $A<1(A>1 )$ demonstrates that surface velocity is more(less) than the free stream velocity, while $A=1$ implies that the surface and free stream velocities are equivalent. The velocity profile decreases (rises) when the values of $A$ are expanded in the first solution on the shrinking (stretching) surface. On the other hand, increasing and decreasing behaviors are seen in the second solution on both surfaces. Velocity profile for various estimations of the Casson parameter $\beta$ on the shrinking/stretching surface is illustrated in Fig. 3. The velocity boundary layer thickness declines at the higher values of $\beta$ in the first solution over both surfaces due to the resistance created by $\beta$ in the fluid flow. In addition, it is worth to mention that when $\beta \to \infty$, the fluid flow behaves like a simple viscous fluid. In other words, it becomes Newtonian fluid. In the second solution, the fluid velocity is expanded initially and gradually decreased in both surfaces. The impact of Hartmann number $M$ on the profile of velocity is delineated in Fig. 4. Hydro boundary layer becomes thinner and the velocity of the fluid is also deaccelerated in both solutions for the maximum intensity of the magnetic parameter. Physically, this is caused by the expansion of Lorentz force which creates the resistance in the fluid flow inside the boundary. Therefore, the velocity of fluid declines.

**Figure 5:**
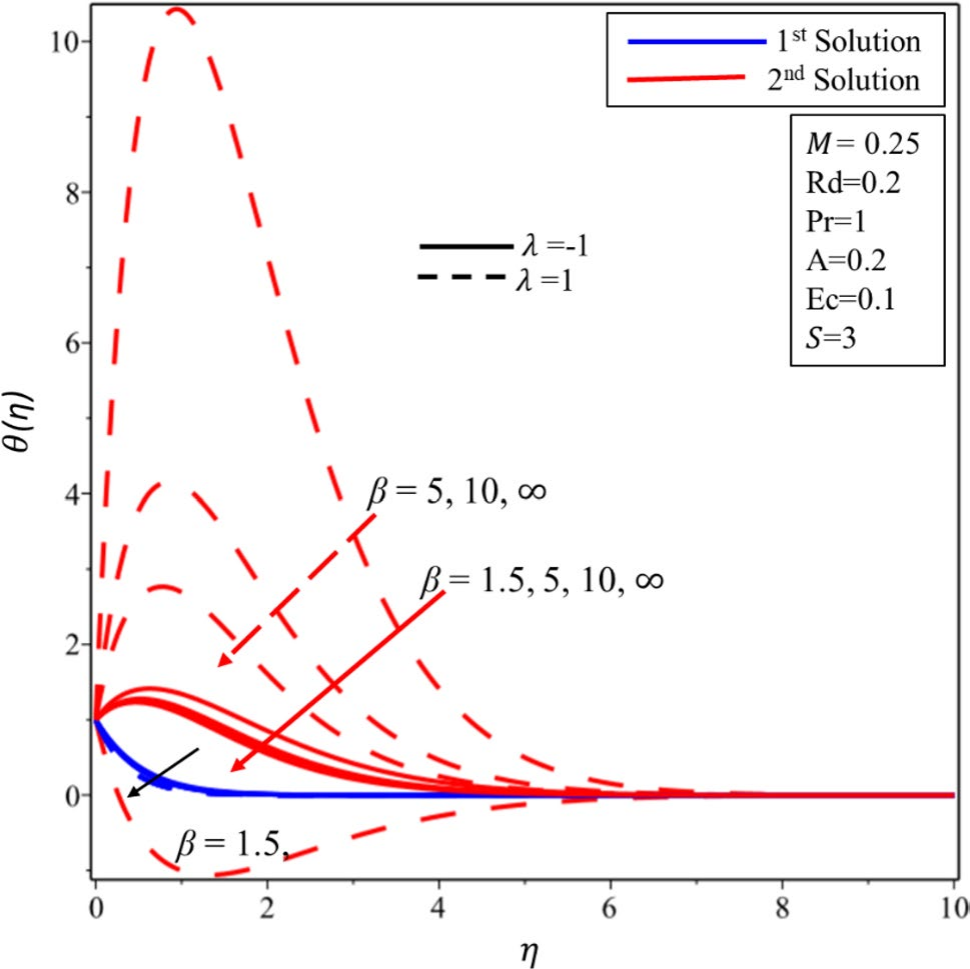
Temperature profile for different values of $\beta$*.*

**Figure 6:**
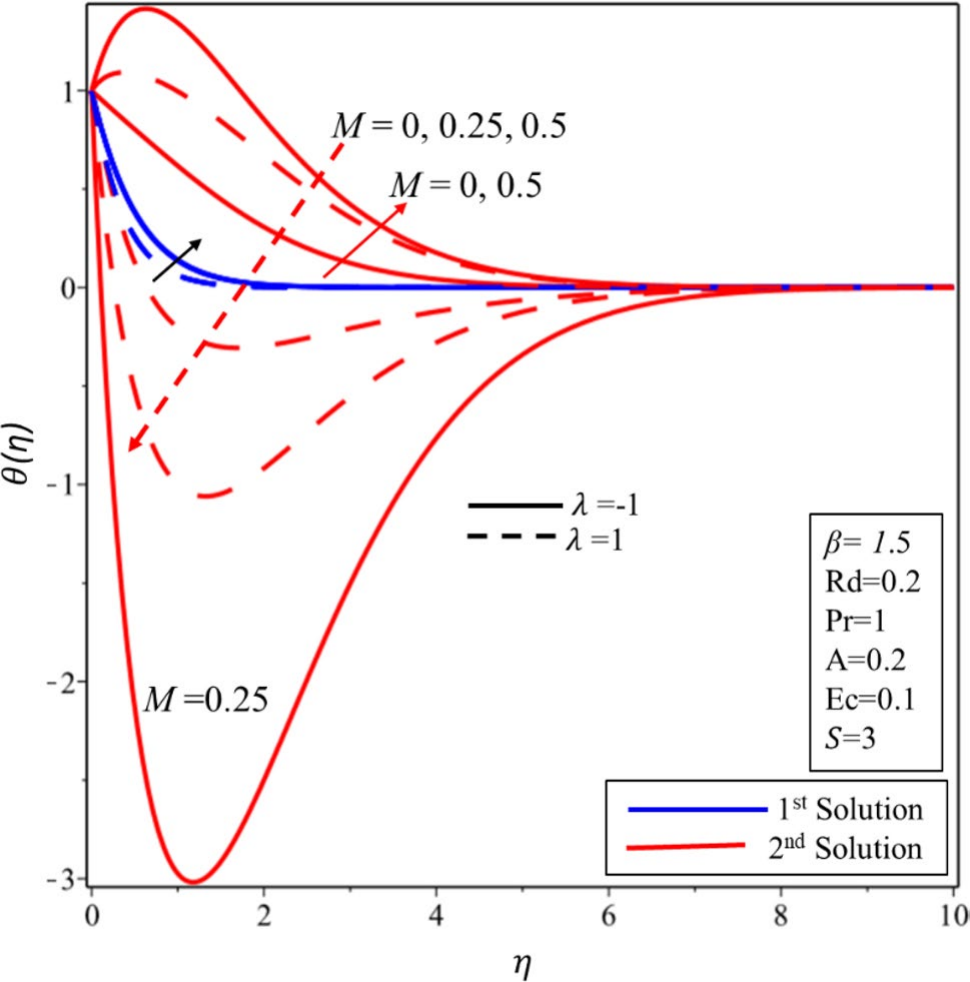
Temperature profile for different values of $M$*.*

**Figure 7:**
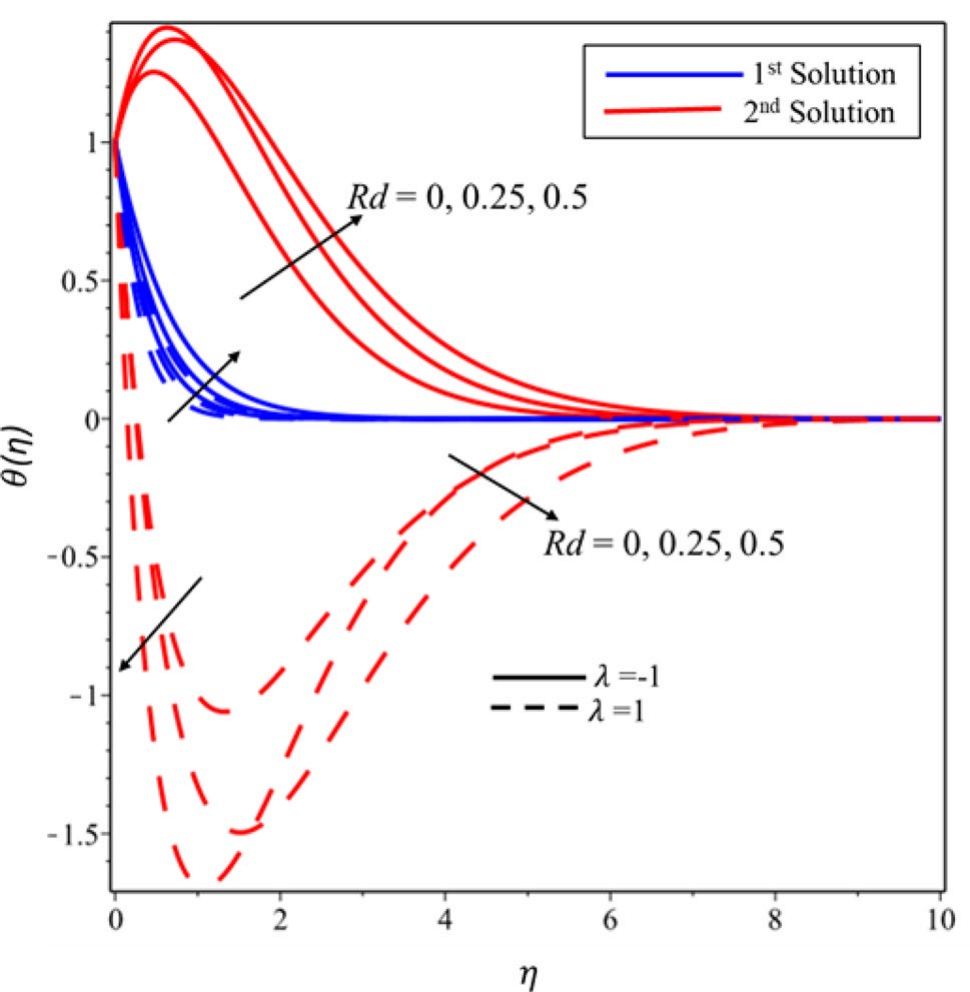
Temperature profile for different values of $Rd$*.*

**Figure 8:**
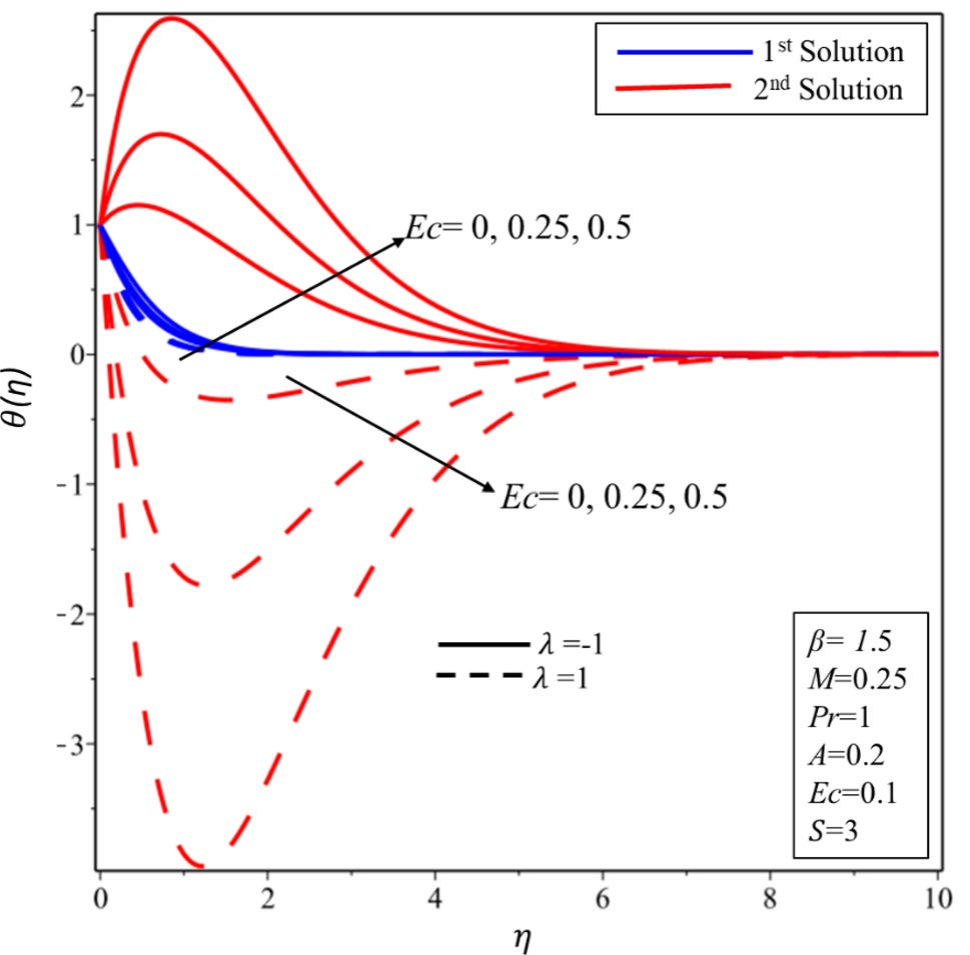
Temperature profile for different values of $Ec$*.*

**Figure 9:**
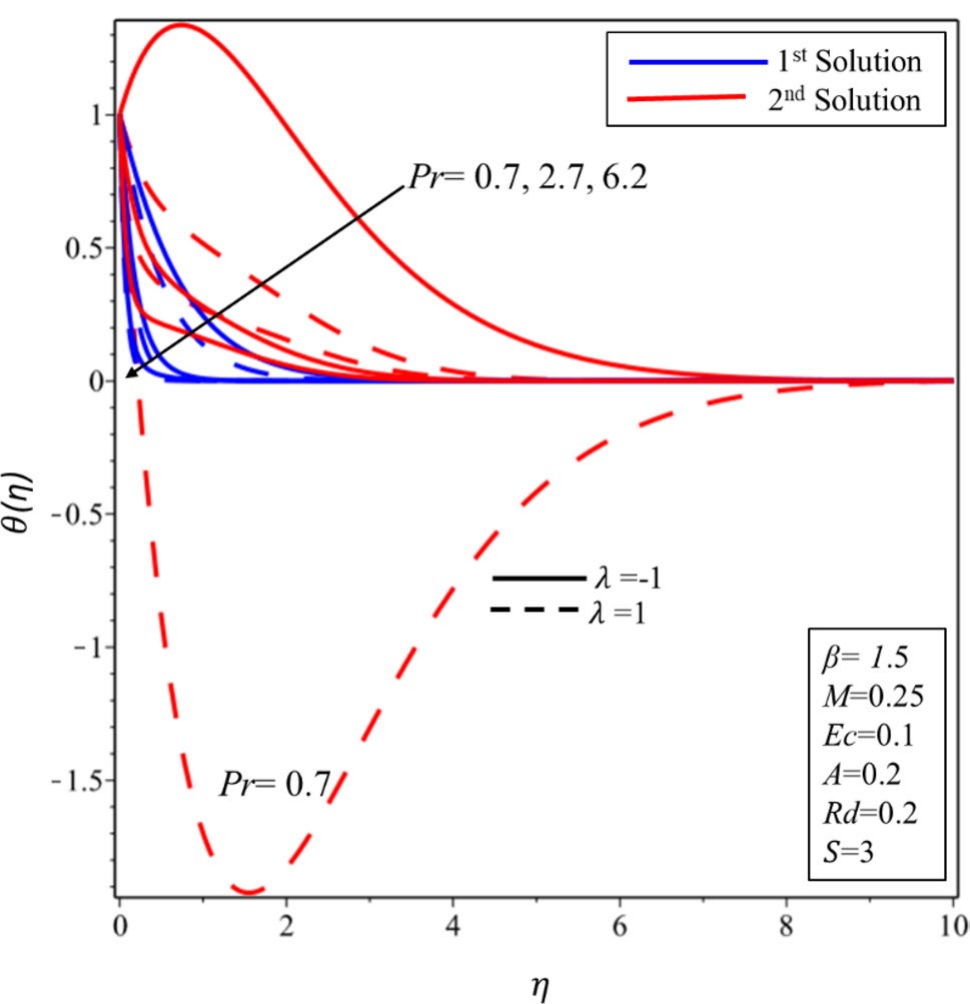
Temperature profile for different values of $Pr$*.*

Figure 5 reveals the relationship between temperature profile and Casson parameter. It has been detected that singularity exists in the temperature distribution in the second solution for the case of stretching surface. Moreover, it is noticed that when Casson parameter is increased then thermal boundary layer and temperature of fluid decrease in both solutions and surfaces. Physically, this reduction is caused by lower values of $\beta$ which is associated to increment (declination) in the yield (shear) stress. The behavior of temperature profile for higher values of $M$ is illustrated in Fig. 6. It is noticed that singularity exists in the range $0<M\le 0.25$ for the case of shrinking surface. This singularity assures us the instability of the second solution and explained in detail in the stability analysis section. It has been found that the fluid’s temperature and thickness of thermal boundary layer rise when magnetic effect enhances for stable and unstable solutions on both surfaces. Physically, it happens due to the fact that higher values of magnetic parameter create a strong force of Lorentz which is an agent to produce more heat from the surface to the fluid. The effect of temperature profile for the thermal radiation was plotted in Fig. 7. The temperature of fluid enhances in both solutions and surfaces for the higher values of $Rd$. Figure 8 exposes the temperature profile for the various values of $Ec$. It is also found that the temperature and thickness of thermal boundary layer increase in both solutions and surfaces as well when viscous dissipation impact is increased in the form of the Eckert number. It is worth mentioning that $Ec<<1$ indicates the balanced between convection and conduction in the energy equation. Practically, the increments in the temperature profiles can be explained as “the reduction of the boundary layer enthalpy difference for advanced values of Eckert number” ^47^. Figure 9 displays the nature of temperature profile for the increasing values of the Prandtl number $Pr.$ It is noticed that thinner thermal boundary is for the larger values of the Prandtl number in both solutions and surfaces. In addition, there exists singularity in the unstable solution for the stretching case. Moreover, this reduction in temperature and thickness of the thermal boundary layer is affected by the lower thermal diffusivity since the relationship between the $Pr$ with thermal diffusivity is reciprocal to each other. Henceforth, the temperature of fluid decreases for the higher values of the Prandtl number.

**Figure 10:**
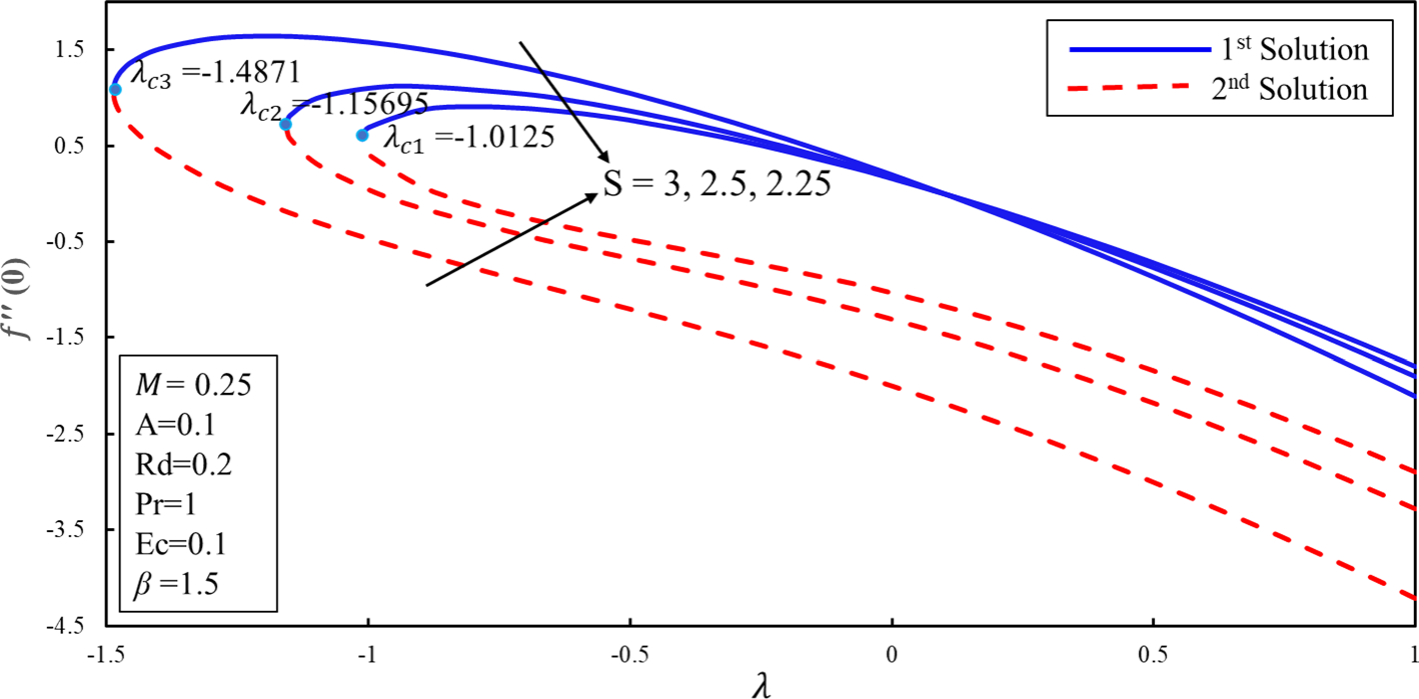
Coefficient of skin friction for different values of $S$*.*

**Figure 11:**
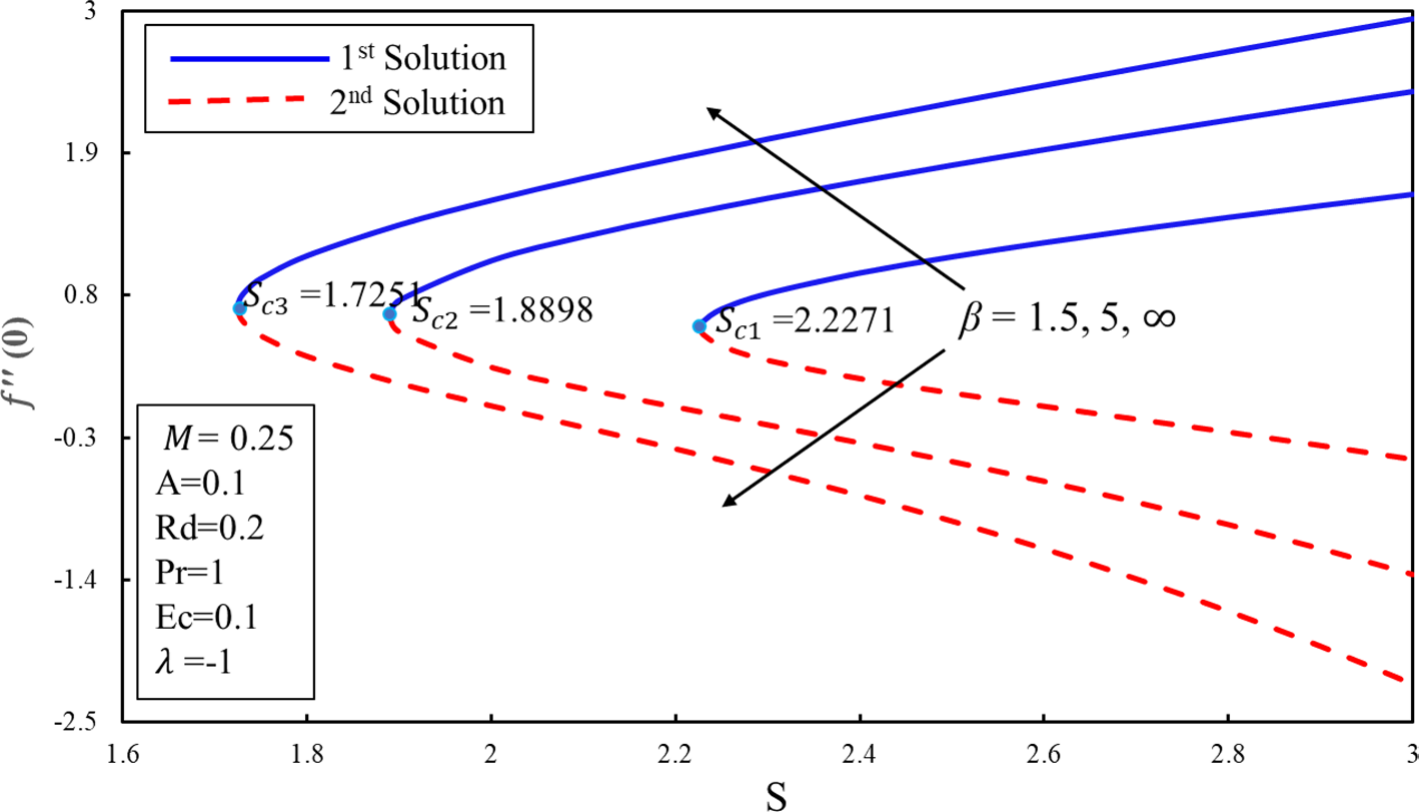
Coefficient of skin friction for different values of $A$*.*

**Figure 12:**
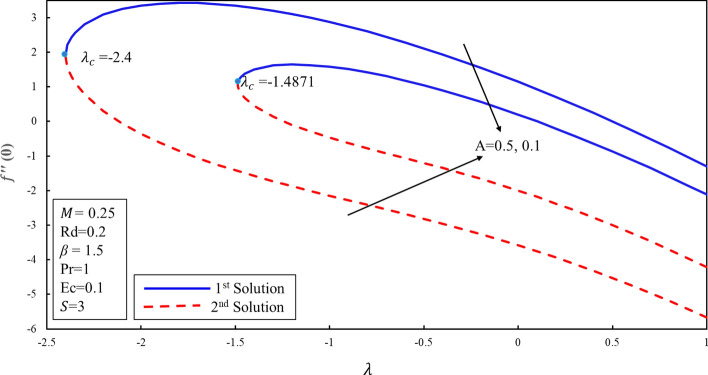
Coefficient of skin friction for different values of $\beta$*.*

**Figure 13:**
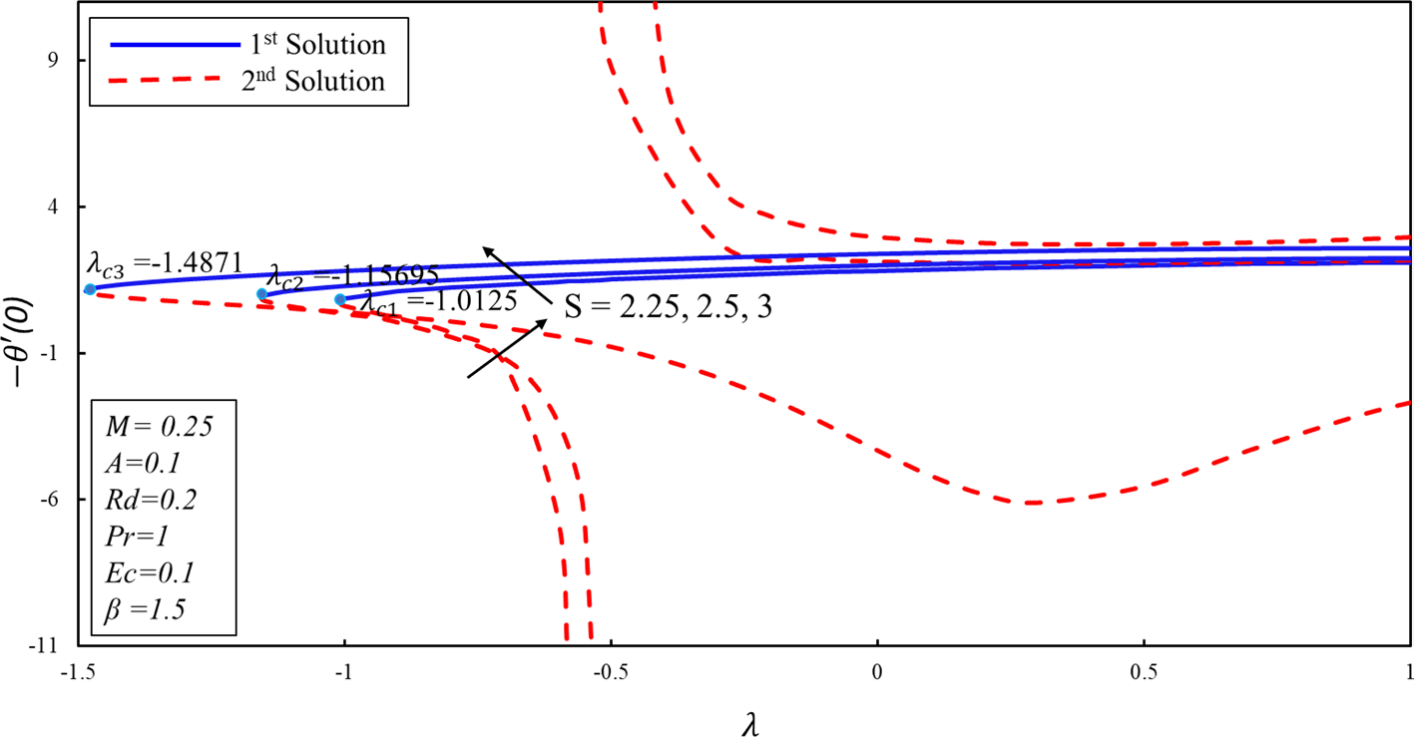
Rate of heat transfer for different values of $S$*.*

**Figure 14:**
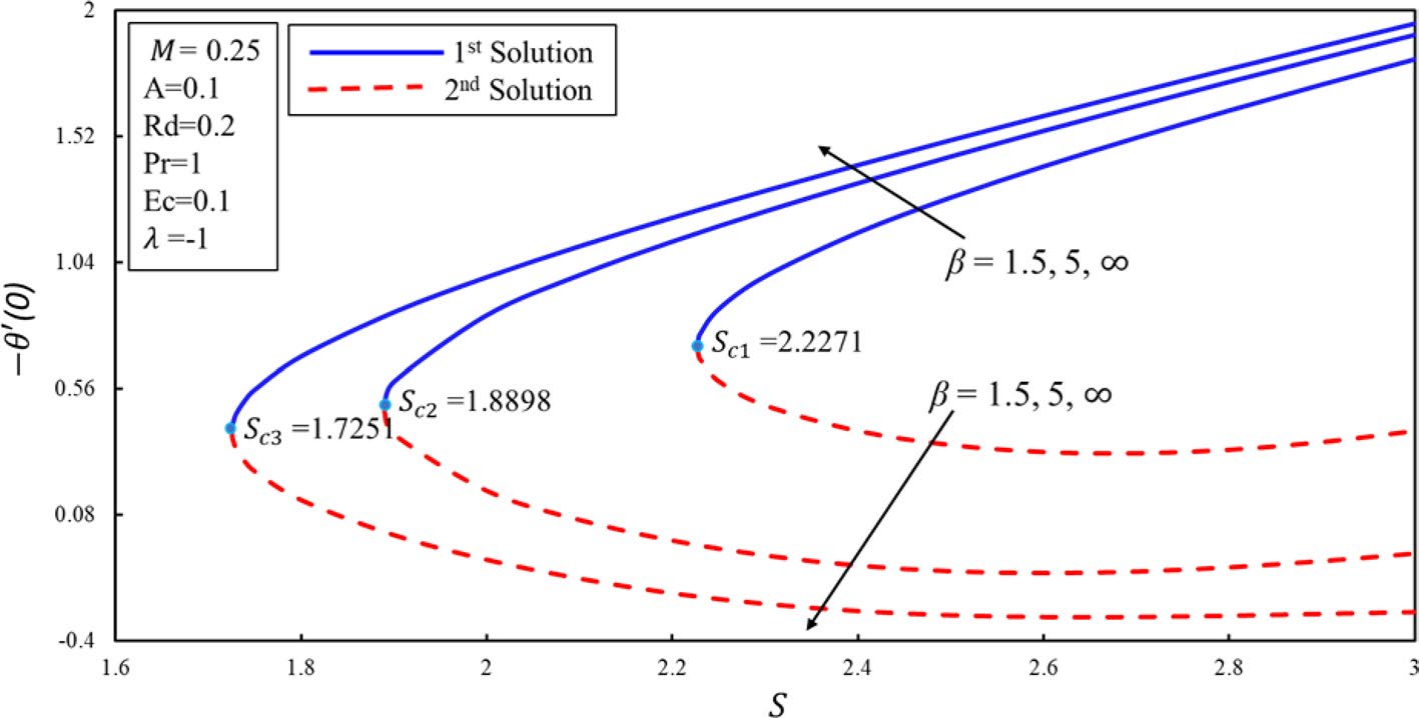
Rate of heat transfer for different values of $\upbeta$.

Figure 10 displays the correlation between the skin friction coefficients with λ and $S$. It is analyzed that the reduction in the suction lowers the skin friction in both solutions which infers that the contact of surface with molecules of fluid is decreasing for the lower effect of the suction. Moreover, an interesting behavior is observed in the first solution where the skin friction rises for the lower values of the suction over the stretching surface. It is also noticed that dual solutions exist on both surfaces. Further, there are the ranges of the dual solutions ($\lambda \ge {\lambda }_{ci}$) and no-solution ($\lambda <{\lambda }_{ci}$) which depend upon the values of ${\lambda }_{ci}$ where $i=\mathrm{1,2},3$. The same behavior is also noticed in Fig. 11. It is worth mentioning that stagnation point $A$ has direct relationship with the skin friction coefficient. Figure 12 demonstrates the impact of higher values of $\beta$ on $f\prime\prime\left(0\right)$. It is examined that $f\prime\prime\left(0\right)$ is lower for the higher values of the non-Newtonian parameter in the first solution. This decreasing behavior of $f\prime\prime(0)$ is due to the inverse relation of shear stress and the yield stress in the fluid equations. On contrary, the reverse behavior is noted for the higher values of $\beta$ in the second solution. Further, increments in the suction produce more (less) drag force in the first (second) solution. It is also found that no solution exists when $S<{S}_{ci}$ while dual solution obtained when for $i=\mathrm{1,2},3$. Figure 13 describes the behavior of heat transfer rate for different values of velocity ratio parameter $\lambda$. There are multiple singularities occur in the unstable solution which demonstrate the instability of the flow for the second solution. Moreover, the rate of heat transfer enhances for the high effect of the suction in the first solution. Finally, the temperature gradient was plotted in Fig. 14. The rate of heat transfer is advanced for the more noteworthy estimations of mass suction in the first solution. Then again, the turnaround pattern is perceived for the second solution.

## Conclusion

The rate of heat transfer of the steady MHD stagnation point flow of Casson fluid on the shrinking/stretching surface in the existence of thermal radiation and viscous dissipation has been examined. By using similarity transformation, the self-similar nonlinear ODEs have been gotten and then solved by using the shooting method in MAPLE software. Double solutions occur for the different ranges of the velocity ratio parameter and mass suction parameter. Stability analysis is done for the solutions by using BVP4C solver in the MATLAB software and the results suggest that only the first solution is the stable. It is found that the velocity and its boundary layer thickness decrease for the greater $A$ in the first solution on the shrinking surface. The thickness of the thermal boundary enhances for the advanced values of the Eckert number and thermal radiation parameter while opposite behavior of temperature profile is noticed for the higher values of the Prandtl number in both solutions. The coefficient of skin friction is reduced by the velocity ratio parameter for both solutions. The coefficient of skin friction decreases for the wall mass transfer parameter in the second solution. In the first solution, however, the behavior of the skin friction contradicts with the second solution.
